# Mosaic Tetrasomy of 9p24.3q21.11 postnatally identified in an infant born with multiple congenital malformations: a case report

**DOI:** 10.1186/s12887-018-1275-8

**Published:** 2018-09-07

**Authors:** Irene Plaza Pinto, Lysa Bernardes Minasi, Raphael Steckelberg, Claudio Carlos da Silva, Aparecido Divino da Cruz

**Affiliations:** 1 Biotechnology and Biodiversity PhD Program, Federal University of Goias, Rede Centro Oeste de Pós-Graduação de Pesquisa e Inovação, Rua 235, n. 40, Setor Leste Universitário, Goiânia, GO 74605-050 Brazil; 2 Replicon Research Group, Department of Biology, Pontifical Catholic University of Goias, Rua 235, n. 40, Setor Leste Universitário, Goiânia, GO 74605-050 Brazil; 3 Genetics Master’s Program, Pontifical Catholic University of Goias, Rua 235, n. 40, Setor Leste Universitário, Goiânia, GO 74605-050 Brazil; 4 Maternity Hospital Amparo, Av T-12 n° 280 Setor Bueno, Goiânia, GO Brazil; 5 Dr Henrique Santillo Rehabilitation and Readjustment Center, Av. Ver. José Monteiro, 1655, Setor Negrão de Lima, Goiânia, GO Brazil; 6 Human Cytogenetics and Molecular Genetics Laboratory, Secretary of Goias State for Public Health, Goiânia, GO Brazil; 7 State University of Goias, Eseffego, Goiânia, Goiás Brazil

**Keywords:** SMC, Chromosome 9, Congenital disorders, Genetics, CMA

## Abstract

**Background:**

Supernumerary Marker Chromosomes consist in structurally abnormal chromosomes, considered as an extra chromosome in which around 70% occur as a de novo event and about 30% of the cases are mosaic. Tetrasomy 9p is a rare chromosomal abnormality described as the presence of a supernumerary isochromosome 9p. Clinical features of tetrasomy 9p include a variety of physical and developmental abnormalities.

**Case presentation:**

Herein, we reported a postnatal case of a newborn who died in early infancy with multiple congenital malformations due to a mosaic de novo tetrasomy 9p detected by Chromosomal Microarray Analysis. Conventional cytogenetics analysis of the proband was 47,XY,+mar[45]/46,XY[5]. The parental karyotypes presented no visible numerical or structural alterations. Microarray Analysis of the proband revealed that the marker chromosome corresponded to a mosaic de novo gain at 9p24.3q21.11.

**Conclusions:**

Chromosomal Microarray Analysis was helpful to identify the origin of the supernumerary marker chromosome and it was a powerful tool to carry out genetic diagnostic, guiding the medical diagnosis. Furthermore, the CMA allowed observing at the first time in Central Brazil the tetrasomy 9p and partial tetrasomy 9q in mosaic, encompassing a large duplicated region with several morbid genes, in an infant with multiple congenital malformations.

## Background

Supernumerary Marker Chromosomes (SMC) are structurally abnormal chromosomes whose origin cannot to be adequately established by G banding karyotyping [1, 2]. In general, SMC are seen as an extra chromosome in metaphase spreads with an incidence rate of 0.43/1000 in postnatal cases and about 1/1000 in prenatal testing. Approximately 70% of SMC occur de novo and about 30% of the cases are mosaic [3, 4].

Tetrasomy 9p is a rare chromosomal abnormality described as the presence of a supernumerary isochromosome 9p, initially found as a SMC. Further investigation of the SMC commonly shows involvement of the entire 9p, or the entire 9p with part of the heterochromatic region of 9q, or yet the entire 9p with heterochromatic region of 9q, and part of the euchromatic material of 9q [5, 6]. Moreover, around 30% of known cases of tetrasomy 9p exhibits chromosome mosaicism. Both constitutive and mosaic SMC of chromosome 9 comprises a clinically noticeable syndrome [3, 7, 8].

Clinical phenotype of tetrasomy 9p includes a variety of physical and developmental abnormalities. Commonly, patients have distinctive facial appearances with hypertelorism, cleft lip or palate, ear anomalies, and micrognathia [3, 9]. In addition, recurrent clinical features include developmental delay, central nervous system anomaly, limb defects, postnatal growth failure, congenital heart disease, renal anomalies, and short neck with excess nuchal skin [8].

Herein, we report a postnatal case of a newborn who had multiple congenital malformations due to a mosaic de novo tetrasomy of 9p and partial tetrasomy of 9q detected by Chromosomal Microarray Analysis (CMA).

## Case presentation

**Fig. 1:**
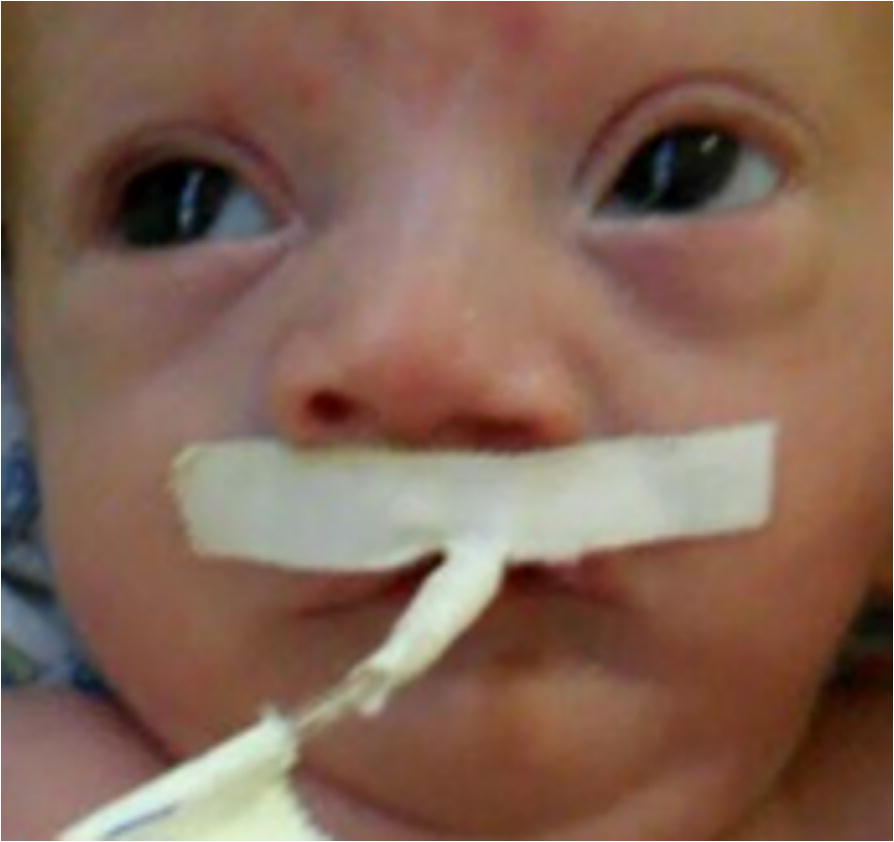
Dismorphological facial features of newborn with multiple congenital malformations from Central Brazil

The proband was a preterm newborn boy, the first child of non-consanguineous parents, born at 31 weeks gestation to a 44-year old father and a 43-year old mother by cesarean section. At birth, the child weighed 1,480 g, measured 44 cm in crown-to-heel length, and exhibited multiple congenital anomalies. The newborn was transferred to the Intensive Care Units (ICU) immediately after birth. His general health condition deteriorated progressively, leading to his death at 105th days after birth. The newborn had brain malformation, including ventriculomegaly and corpus callosum dysgenesis, cleft lip and palate, retrognathism, hypertelorism, clenched hands with overlapping fingers, and hypotonia. Additionally, he revealed mild heart septal hypertrophy, ambiguous genitalia, enlarged kidneys without corticomedullary differentiation, and gallbladder with tiny cystic formations (Fig. 1). His mother had three miscarriages from previous marriages and one miscarriage with her current husband. The remaining of his family history was otherwise unremarkable.

Both parents signed a written informed consent and the mother signed as the legal representative for the child. Peripheral blood was obtained to isolate genomic DNA for CMA using Qiagen QIAamp® DNA Mini kit (Hilden, Germany). Karyotyping was performed in a private laboratory through conventional cell culture, harvesting, and GTG banding with a > 550 bands resolution following standard procedures [10]. Chromosome analyses were done using Zeiss Axio Scope (Jena, Germany) and the software IKAROS® (Metasystems Corporation, Altlussheim, Germany). All laboratory procedures were carried out following international standardized protocols and consensual criteria of quality.

The CMA was carried out on proband and his biological parents using the GeneChip® CytoScanHD™ (Affymetrix, Santa Clara, USA) following the manufacturer’s recommendations without modifications. Chromosomal analyses were done using the Chromosome Analysis Suite (ChAS®) software (Affymetrix, Santa Clara, USA) and the CNVs found in the patient were analyzed in comparison with public databases, including Database of Genomic Variants (DGV), Database of Chromosomal Imbalance and Phenotype in Humans using Ensemble Resources (DECIPHER), and CytoScanHD™ Array Database. Furthermore, CNVs were classified according to their nature, based on [11, 12].

**Fig. 2:**
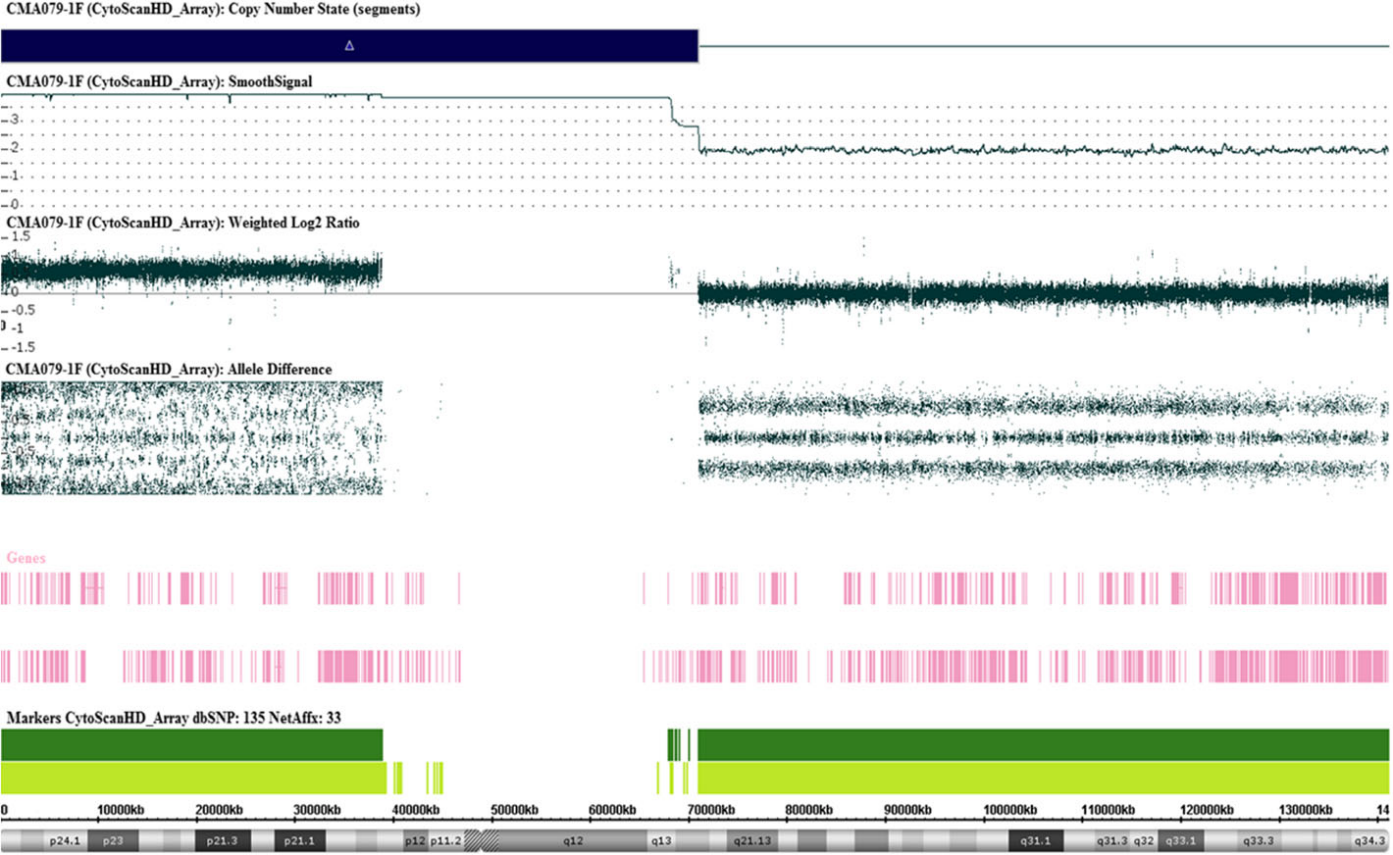
ChAS® (Affymetrix, USA) interface indicating the results of Chromosomal Microarray Analysis showing the derivative chromosome 9 responsible for tetrasomy 9p extended distally to 9q21.11

The proband showed a male karyotype with a large submetacentric SMC in 90% of the analyzed metaphases after counting 50 metaphase spreads. His karyotype was 47,XY,+mar[45]/46,XY[5], suggesting 10% mosaicism. The parental karyotypes and CMA results had no visible numerical or structural alterations. The proband’s CMA revealed the marker chromosome corresponded to a de novo 70.77 Mb gain at arr[GRCh37] 9p24.3q21.11(203,861_70,974,662)× 4[0.3] dn with 30% mosaicism, encompassing 286 genes, including 152 OMIM morbid genes (Fig. 2).

## Discussion and conclusions

Chromosomal alterations associated with a spectrum of multiple congenital anomalies are most frequently numerical and are identified in 0.3–1% of newborns [13, 14]. Partial tetrasomy 9 is not yet a well recognizable clinical syndrome due to the limited number of postnatal cases described to date. Moreover, patients with partial tetrasomy 9 have variable phenotypes depending on the size and position of the duplicated region and the degree of mosaicism [6–8, 15].

Here we reported a case of a boy who died postneonataly in early infancy whose karyotype indicated an additional SMC which origin could not be determined by GTG-banding. Microarray analysis showed 47,XY,+mar[45]/46,XY[5].arr[GRCh37] 9p24.3q21.11(203,861_70,974,662)× 4[0.3] dn. Copy Number Variations (CNVs) within the region of gain indicated tetrasomy, revealing full tetrasomy 9p and partial tetrasomy 9q, including the proximal euchromatic region of the long arm in a mosaic state. Parental CMA were uneventful.

According to [4], about of 40% of all SMCs are derived from non-acrocentric autosomes and the risk of an abnormal phenotype is approximately 28%. Mosaic tetrasomy of 9p most commonly arises as a de novo chromosomal rearrangement resulting from an early sporadic error in embryonic development. Thus, the risk of recurrence is relatively low and this has important implications in genetic counseling for the family [16]. Furthermore, it is important to emphasize the mother history of miscarriage because either a cryptic translocation or some aberrant chromosomal constitution restricted to the mother’s gonads, most likely as a gonadal mosaicism, could be responsible for her failure to conceive health offspring. However, the family refused to undergo genetic counseling and refused any additional genetic testing.

Tetrasomy 9p is a rare disorder firstly described by [17]. Up to now, at least 34 postnatal cases have been previously reported in medical literature, varying greatly in chromosomal range and phenotype severity. Although the affected population is small, males seemed to be slightly more affected than females [5]. The clinical severity of tetrasomy 9p varies from neonatal death to mild developmental delay and minor anomalies [9, 18, 19]. Furthermore, when the tetrasomic segment extend to 9q22-9q32 it is common to observe intrauterine growth retardation, skeletal anomalies, cleft lip/palate, and heart defects [15].

According to some authors, three different types of supernumerary isochromosome 9p have been described so far. There are tetrasomies containing exclusively the entire 9p, some includes the entire 9p and a small proportion of the heterochromatic region of 9q, and yet some harbors the entire 9p and a large portion of 9q extending to 9q21, including both heterochromatic and euchromatic regions of 9q [3, 5, 6]. The tetrasomy was categorized as the third type of SMC involving chromosome 9, with breakpoints at 9q21.11 and isochromosome 9p, justifying the severity of symptoms in the studied proband.

There is a positive correlation between degree of mosaicism and the severity of the phenotype in infants having tetrasomy 9p. However, the breakpoint of 9q extended distally to 9q21.11, which encompasses a large duplicated region with several OMIM morbid genes. This finding supports the claim the nature of the involved genes was more crucial than the level of mosaicism in defining phenotype’s severity in our proband.

The CMA was helpful to identify the origin of the SMC in the proband born to parents with normal karyotypes. In addition, it was an effective method to identify the tetrasomy 9p and partial tetrasomy 9q in mosaic in an infant with multiple congenital malformations for the first time in Central Brazil. Rare and complex phenotypes must always be investigated to define subsets and allow the phenotype/genotype correlation. Furthermore, it was recommended to the family to undergo a non-directive genetic counseling to help understanding the familial implications of genetic contribution to disease and the chance of recurrence.

## Abbreviations

ChAS: Chromosome Analysis Suite
CMA: Chromosomal Microarray Analysis
CNVs: Copy Number Variations
DECIPHER: Database of Chromosomal Imbalance and Phenotype in Humans using Ensemble Resources
DGV: Database of Genomic Variants
ICU: Intensive Care Units
OMIM: Online Mendenlian Inheritance in Man
SMC: Supernumerary Marker Chromosomes
SNP: Single Nucleotide Polymorphism
